# NETWORKING MUSEUMS, OLDER PEOPLE, AND COMMUNITIES: UNCOVERING AND SUSTAINING STRENGTHS IN AGING

**DOI:** 10.1093/geroni/igz038.1322

**Published:** 2019-11-08

**Authors:** Teresa Bonner

**Affiliations:** Aroha Philanthropies, Minneapolis, Minnesota, United States

## Abstract

Museums, like other cultural institutions, are beginning to embrace a new role: facilitating creativity of older adults through education programs. A cohort of 20 American museums from Alaska to Puerto Rico are embarking on an ambitious two-year program, funded by Aroha Philanthropies, to develop successful creative aging programs in their communities. The cohort includes art museums, history centers, botanical gardens and a science museum. The group has received extensive training and technical assistance to build their capacity and awareness of needs and desires of older adults. The American Alliance of Museums (AAM), a partner in this initiative, is leading a deep dive into the potential of museums to enable older adults to learn, make and share the arts. With Aroha’s support, AAM has created a two-year position, the Aroha Senior Fellowship in Museums and Creative Aging to lead an exploration of how museums can deepen their engagement with creative aging.

